# 1,4-Dibromo-2,5-bis­(hex­yloxy)benzene

**DOI:** 10.1107/S1600536808028730

**Published:** 2008-09-13

**Authors:** Ying-Fei Li, Chen Xu, Fei-Fei Cen, Zhi-Qiang Wang, Yu-Qing Zhang

**Affiliations:** a Chemical Engineering and Pharmaceutics School, Henan University of Science and Technology, Luoyang 471003, People’s Republic of China; b College of Chemistry and Chemical Engineering, Luoyang Normal University, Luoyang 471022, People’s Republic of China

## Abstract

In the centrosymmetric title compound, C_18_H_28_Br_2_O_2_, the alkyl chains adopt a fully extended all-*trans* conformation and each of them is almost planar. In addition, the alkyl chains are coplanar with the benzene ring. Inter­molecular Br⋯Br inter­actions [3.410 (3) Å] are present, resulting in a one-dimensional supra­molecular architecture.

## Related literature

For related literature, see: Ali *et al.* (2008 ▶); Brammer (2004 ▶); Desiraju & Parthasarathy (1989 ▶); Kuriger *et al.* (2008 ▶); Maruyama & Kawanishi (2002 ▶).
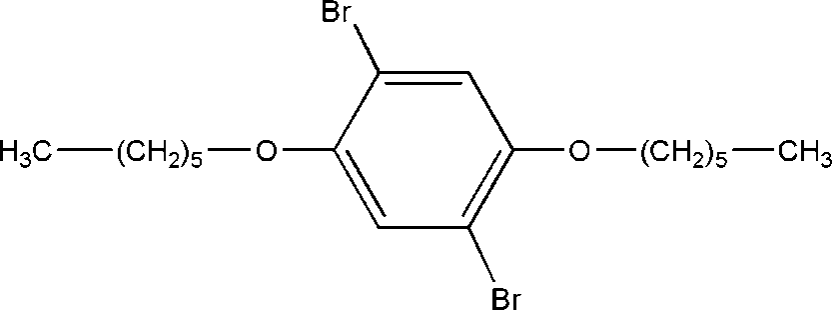


## Experimental

### 

#### Crystal data

C_18_H_28_Br_2_O_2_
*M* *_r_* = 436.22
Triclinic, 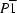

*a* = 6.9638 (12) Å
*b* = 8.2581 (14) Å
*c* = 9.7321 (17) Å
α = 107.012 (2)°
β = 106.981 (2)°
γ = 99.193 (2)°
*V* = 493.11 (15) Å^3^
*Z* = 1
Mo *K*α radiation
μ = 4.12 mm^−1^
*T* = 295 (2) K
0.28 × 0.27 × 0.07 mm

#### Data collection

Bruker SMART CCD diffractometer
Absorption correction: multi-scan (*SADABS*; Sheldrick, 1996 ▶) *T*_min_ = 0.391, *T*_max_ = 0.764
3675 measured reflections
1818 independent reflections
1567 reflections with *I* > 2σ(*I*)
*R*_int_ = 0.018

#### Refinement

*R*[*F* ^2^ > 2σ(*F* ^2^)] = 0.025
*wR*(*F* ^2^) = 0.063
*S* = 1.06
1818 reflections
101 parameters
H-atom parameters constrained
Δρ_max_ = 0.31 e Å^−3^
Δρ_min_ = −0.23 e Å^−3^

### 

Data collection: *SMART* (Bruker, 2004 ▶); cell refinement: *SAINT* (Bruker, 2004 ▶); data reduction: *SAINT*; program(s) used to solve structure: *SHELXS97* (Sheldrick, 2008 ▶); program(s) used to refine structure: *SHELXL97* (Sheldrick, 2008 ▶); molecular graphics: *SHELXTL* (Sheldrick, 2008 ▶); software used to prepare material for publication: *SHELXTL*.

## Supplementary Material

Crystal structure: contains datablocks global, I. DOI: 10.1107/S1600536808028730/si2104sup1.cif

Structure factors: contains datablocks I. DOI: 10.1107/S1600536808028730/si2104Isup2.hkl

Additional supplementary materials:  crystallographic information; 3D view; checkCIF report

## Figures and Tables

**Fig. 1.**
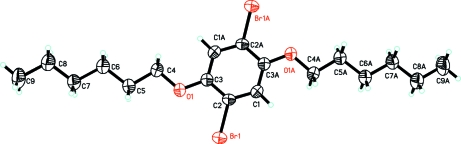
The molecular structure of the title compound with displacement ellipsoids at the 50% probability level. Inversion related atoms are labelled with an A. (Symmetry code: -x, 1 - y, -z).

**Fig. 2.**
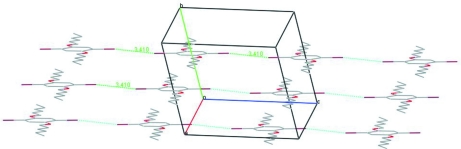
Partial view of the crystal packing showing the formation of the infinite chains of molecules formed by the intermolecular Br···Br interactions. Intercalated neighboring chains complete the sheets in the structure running parallel to (100). H atoms have been omitted for clarity.

## supplementary crystallographic information

### Comment

Noncovalent interactions play an important role in designing superstructures (Brammer, 2004). Among these weak forces, the intermolecular interactions between halogen atoms have been a subject of interest (Desiraju *et al.*, 1989). In order to gain more insight into the structure-regulating ability of intermolecular Br···Br interactions, herein we report the crystal structure of the title compound.

A view of the centrosymmetric molecular structure of the title compound is given in Fig.1. The alkyl chains are in the fully extended all-*trans* conformation and each of them is almost perfectly planar. The C—C—O—C torsion angles of 3.4 (4)*^o^*, indicate that the two alkyl chains are coplanar with the benzene ring. The crystal structure of the titile compound reveals the presence of a near linear C—Br···Br fragment[C—Br···Br=155.6 (3)*^o^*], the Br···Br distance (3.410 Å) is shorter than the sum of van der Waals radii(3.72 Å) and those in the other compound [3.634 (4)–3.9527 (9) Å](Kuriger *et al.*, 2008; Ali *et al.*, 2008). Owing to the intermolecular Br···Br interactions, the crystal structure of the title compound is extended to a one-dimensional chain structure. The chains are intercalated by van der Waals forces (Fig.2).

### Experimental

The title compound was prepared as described in literature (Maruyama & Kawanishi 2002) and recrystallized from dichloromethane-ethanol at room temperature to give the desired crystals suitable for single-crystal X-ray diffraction.

### Refinement

H atoms attached to C atoms of the title compound were placed in geometrically idealized positions and treated as riding with C—H distances constrained to 0.93 (aromatic CH), or 0.96 Å (methyl CH3), and 0.97 Å (methylene CH2) and constrained to ride on their parent atoms, with *U*ĩso~(H) = 1.2Ueq(C)(1.5Ueq for methyl H).

### Crystal data

**Table:**

C_18_H_28_Br_2_O_2_	*Z* = 1
*M**_r_* = 436.22	*F*(000) = 222
Triclinic, *P*1	*D*_x_ = 1.469 Mg m^−^^3^
*a* = 6.9638 (12) Å	Mo *K*α radiation, λ = 0.71073 Å
*b* = 8.2581 (14) Å	Cell parameters from 1772 reflections
*c* = 9.7321 (17) Å	θ = 2.3–26.0°
α = 107.012 (2)°	µ = 4.12 mm^−^^1^
β = 106.981 (2)°	*T* = 295 K
γ = 99.193 (2)°	Block, colourless
*V* = 493.11 (15) Å^3^	0.28 × 0.27 × 0.07 mm

### Data collection

**Table:**

Bruker SMART CCD diffractometer	1818 independent reflections
Radiation source: fine-focus sealed tube	1567 reflections with *I* > 2σ(*I*)
graphite	*R*_int_ = 0.018
phi and ω scans	θ_max_ = 25.5°, θ_min_ = 2.7°
Absorption correction: multi-scan (SADABS; Sheldrick, 1996)	*h* = −8→8
*T*_min_ = 0.391, *T*_max_ = 0.764	*k* = −9→9
3675 measured reflections	*l* = −11→11

### Refinement

**Table:**

Refinement on *F*^2^	Primary atom site location: structure-invariant direct methods
Least-squares matrix: full	Secondary atom site location: difference Fourier map
*R*[*F*^2^ > 2σ(*F*^2^)] = 0.025	Hydrogen site location: inferred from neighbouring sites
*wR*(*F*^2^) = 0.063	H-atom parameters constrained
*S* = 1.06	*w* = 1/[σ^2^(*F*_o_^2^) + (0.0346*P*)^2^ + 0.0096*P*] where *P* = (*F*_o_^2^ + 2*F*_c_^2^)/3
1818 reflections	(Δ/σ)_max_ = 0.001
101 parameters	Δρ_max_ = 0.31 e Å^−^^3^
0 restraints	Δρ_min_ = −0.23 e Å^−^^3^

### Special details

**Table:**

Geometry. All e.s.d.'s (except the e.s.d. in the dihedral angle between two l.s. planes) are estimated using the full covariance matrix. The cell e.s.d.'s are taken into account individually in the estimation of e.s.d.'s in distances, angles and torsion angles; correlations between e.s.d.'s in cell parameters are only used when they are defined by crystal symmetry. An approximate (isotropic) treatment of cell e.s.d.'s is used for estimating e.s.d.'s involving l.s. planes.
Refinement. Refinement of *F*^2^ against ALL reflections. The weighted *R*-factor *wR* and goodness of fit *S* are based on *F*^2^, conventional *R*-factors *R* are based on *F*, with *F* set to zero for negative *F*^2^. The threshold expression of *F*^2^ > σ(*F*^2^) is used only for calculating *R*-factors(gt) *etc*. and is not relevant to the choice of reflections for refinement. *R*-factors based on *F*^2^ are statistically about twice as large as those based on *F*, and *R*- factors based on ALL data will be even larger.

### Fractional atomic coordinates and isotropic or equivalent isotropic displacement parameters (Å^2^)

**Table:**

	*x*	*y*	*z*	*U*_iso_*/*U*_eq_	
Br1	0.07253 (4)	0.51292 (4)	−0.31314 (3)	0.05768 (13)	
O1	0.3731 (2)	0.6878 (2)	0.00755 (19)	0.0577 (5)	
C1	−0.1596 (3)	0.4127 (3)	−0.1410 (3)	0.0455 (6)	
H1	−0.2656	0.3549	−0.2366	0.055*	
C2	0.0292 (3)	0.5069 (3)	−0.1316 (3)	0.0418 (5)	
C3	0.1932 (3)	0.5970 (3)	0.0107 (3)	0.0426 (5)	
C4	0.5450 (3)	0.7726 (4)	0.1511 (3)	0.0513 (6)	
H4A	0.5871	0.6864	0.1944	0.062*	
H4B	0.5056	0.8560	0.2240	0.062*	
C5	0.7229 (3)	0.8672 (3)	0.1183 (3)	0.0528 (6)	
H5A	0.6775	0.9504	0.0722	0.063*	
H5B	0.7610	0.7824	0.0455	0.063*	
C6	0.9133 (3)	0.9647 (3)	0.2665 (3)	0.0539 (6)	
H6A	0.8755	1.0516	0.3377	0.065*	
H6B	0.9547	0.8817	0.3143	0.065*	
C7	1.0979 (3)	1.0563 (3)	0.2370 (3)	0.0518 (6)	
H7A	1.1351	0.9693	0.1653	0.062*	
H7B	1.0564	1.1394	0.1894	0.062*	
C8	1.2870 (4)	1.1524 (4)	0.3828 (3)	0.0662 (8)	
H8A	1.3355	1.0679	0.4259	0.079*	
H8B	1.2471	1.2326	0.4576	0.079*	
C9	1.4646 (4)	1.2556 (5)	0.3548 (4)	0.0850 (10)	
H9A	1.5032	1.1771	0.2795	0.127*	
H9B	1.5824	1.3105	0.4494	0.127*	
H9C	1.4200	1.3442	0.3179	0.127*	

### Atomic displacement parameters (Å^2^)

**Table:**

	*U*^11^	*U*^22^	*U*^33^	*U*^12^	*U*^13^	*U*^23^
Br1	0.05214 (17)	0.0815 (2)	0.03895 (16)	0.00640 (13)	0.01726 (12)	0.02627 (13)
O1	0.0382 (9)	0.0797 (12)	0.0427 (9)	−0.0082 (8)	0.0094 (7)	0.0224 (9)
C1	0.0369 (12)	0.0546 (14)	0.0350 (12)	0.0026 (10)	0.0057 (9)	0.0142 (11)
C2	0.0414 (12)	0.0529 (14)	0.0330 (11)	0.0089 (10)	0.0135 (10)	0.0199 (10)
C3	0.0354 (11)	0.0501 (13)	0.0392 (12)	0.0049 (10)	0.0115 (10)	0.0168 (11)
C4	0.0368 (12)	0.0633 (16)	0.0417 (13)	−0.0004 (11)	0.0082 (10)	0.0151 (12)
C5	0.0383 (12)	0.0654 (16)	0.0481 (14)	0.0015 (11)	0.0139 (11)	0.0193 (12)
C6	0.0399 (13)	0.0629 (17)	0.0504 (14)	0.0023 (12)	0.0136 (11)	0.0172 (13)
C7	0.0404 (13)	0.0562 (15)	0.0535 (15)	0.0043 (11)	0.0175 (11)	0.0160 (12)
C8	0.0437 (14)	0.0786 (19)	0.0592 (17)	−0.0001 (13)	0.0128 (13)	0.0150 (15)
C9	0.0468 (16)	0.095 (2)	0.086 (2)	−0.0123 (15)	0.0179 (16)	0.0156 (19)

### Geometric parameters (Å, °)

**Table:**

Br1—C2	1.891 (2)	C5—H5B	0.9700
O1—C3	1.367 (2)	C6—C7	1.529 (3)
O1—C4	1.435 (3)	C6—H6A	0.9700
C1—C3^i^	1.377 (3)	C6—H6B	0.9700
C1—C2	1.379 (3)	C7—C8	1.514 (3)
C1—H1	0.9300	C7—H7A	0.9700
C2—C3	1.406 (3)	C7—H7B	0.9700
C3—C1^i^	1.377 (3)	C8—C9	1.525 (4)
C4—C5	1.522 (3)	C8—H8A	0.9700
C4—H4A	0.9700	C8—H8B	0.9700
C4—H4B	0.9700	C9—H9A	0.9600
C5—C6	1.533 (3)	C9—H9B	0.9600
C5—H5A	0.9700	C9—H9C	0.9600
			
C3—O1—C4	117.26 (18)	C7—C6—H6A	109.1
C3^i^—C1—C2	120.9 (2)	C5—C6—H6A	109.1
C3^i^—C1—H1	119.6	C7—C6—H6B	109.1
C2—C1—H1	119.6	C5—C6—H6B	109.1
C1—C2—C3	121.5 (2)	H6A—C6—H6B	107.9
C1—C2—Br1	119.81 (16)	C8—C7—C6	112.7 (2)
C3—C2—Br1	118.73 (16)	C8—C7—H7A	109.1
O1—C3—C1^i^	125.49 (19)	C6—C7—H7A	109.1
O1—C3—C2	116.8 (2)	C8—C7—H7B	109.1
C1^i^—C3—C2	117.70 (19)	C6—C7—H7B	109.1
O1—C4—C5	107.30 (19)	H7A—C7—H7B	107.8
O1—C4—H4A	110.3	C7—C8—C9	112.5 (3)
C5—C4—H4A	110.3	C7—C8—H8A	109.1
O1—C4—H4B	110.3	C9—C8—H8A	109.1
C5—C4—H4B	110.3	C7—C8—H8B	109.1
H4A—C4—H4B	108.5	C9—C8—H8B	109.1
C4—C5—C6	111.0 (2)	H8A—C8—H8B	107.8
C4—C5—H5A	109.4	C8—C9—H9A	109.5
C6—C5—H5A	109.4	C8—C9—H9B	109.5
C4—C5—H5B	109.4	H9A—C9—H9B	109.5
C6—C5—H5B	109.4	C8—C9—H9C	109.5
H5A—C5—H5B	108.0	H9A—C9—H9C	109.5
C7—C6—C5	112.4 (2)	H9B—C9—H9C	109.5
			
C3^i^—C1—C2—C3	−0.4 (4)	Br1—C2—C3—C1^i^	−178.52 (18)
C3^i^—C1—C2—Br1	178.49 (18)	C3—O1—C4—C5	−178.7 (2)
C4—O1—C3—C1^i^	3.4 (4)	O1—C4—C5—C6	179.1 (2)
C4—O1—C3—C2	−176.9 (2)	C4—C5—C6—C7	178.1 (2)
C1—C2—C3—O1	−179.4 (2)	C5—C6—C7—C8	−179.8 (2)
Br1—C2—C3—O1	1.7 (3)	C6—C7—C8—C9	−175.4 (2)
C1—C2—C3—C1^i^	0.4 (4)		

Symmetry codes: (i) −*x*, −*y*+1, −*z*.
